# Modifying the cancer-immune set point using vaccinia virus expressing re-designed interleukin-2

**DOI:** 10.1038/s41467-018-06954-z

**Published:** 2018-11-08

**Authors:** Zuqiang Liu, Yan Ge, Haiyan Wang, Congrong Ma, Mathilde Feist, Songguang Ju, Z. Sheng Guo, David L. Bartlett

**Affiliations:** 1 Department of Surgery, University of Pittsburgh School of Medicine, Pittsburgh, PA 15213 USA; 2 UPMC Hillman Cancer Center, Pittsburgh, PA 15213 USA; 3 Department of Immunology, School of Biology and Basic Medical Sciences, Medical College, Soochow University, Suzhou, 215123 Jiangsu Province China; 4 Department of Surgery, CCM/CVK, Charité—Universitaetsmedizin Berlin, Berlin, 13353 Germany

## Abstract

The complex immune tumour microenvironment requires an equally complex immunotherapy approach, especially when the cancer-immune set point is non-inflamed. Oncolytic viruses expressing immune activating cytokines might optimally modify the immune microenvironment and improve the antitumour effects. In this study, we have explored a variety of IL-2 constructs expressed by a tumour-selective oncolytic vaccinia virus, designed to maintain IL-2 in the tumour microenvironment to reduce systemic toxicity. An IL-2 construct combining a glycosylphosphatidylinositol (GPI) anchor with a rigid peptide linker leads to functional IL-2 expression on the tumour cell surface and in the tumour microenvironment. This virus construct effectively modifies the cancer-immune set point and treats a variety of murine tumour models with no toxic side effects. In combination with PD-1/PD-L1 blockade this virus cures most of the mice with a high tumour burden. This combination represents a treatment for cancers which are to date unresponsive to immunotherapy.

## Abstract (web summary)

IL-2 is used systemically for cancer therapy but it is associated with severe toxicity. Here, the authors design a recombinant vaccinia virus expressing membrane-bound IL-2 that shows therapeutic efficacy alone or in combination with checkpoint inhibitors in colon cancer-bearing mice.

## Introduction

The immune microenvironment for many non-immunogenic tumours lacks neo-antigen expression, co-stimulatory molecules, tumour-specific immune cells, inflammatory infiltrates, and is often replete with immunosuppressive factors^1–3^. Reversing this negative cancer-immune set point is currently the biggest challenge for oncology research^4^. An oncolytic virus replicates selectively in tumour tissue leading to a potent inflammatory response in the tumour microenvironment^5^. As the virus is cleared by the immune system, immune checkpoints function to suppress the inflammatory response. The pleiotropic cytokine interleukin-2 (IL-2) is a potent T-cell mitogen and activator that can extend the function of T-cells in this setting and improve the immune clearance of the tumour^6–9^. IL-2 is used systemically for cancer therapy and is associated with severe toxicity, whereas its effect is best kept within the tumour microenvironment. Considerable efforts have been devoted to developing IL-2 variants to improve its activity and safety^10–19^.

Here, we describe a new form of IL-2 immunotherapy through delivery of a cell membrane-bound IL-2 by tumour-targeted oncolytic vaccinia virus. In addition to the neo-antigen release and inflammatory response from the virus, the IL-2 acts in the tumour microenvironment to expand tumour-specific T-cells while limiting the severe life-threatening side effects associated with systemic IL-2. This construct (vvDD-IL-2-RG) was sufficient to cure the majority of mice with early-stage peritoneal colon cancer, but more advanced disease recurred. Combining vvDD-IL-2-RG with immune checkpoint inhibition leads to a cure in most of the mice with advanced tumours. These findings suggest that a tumour-selective replicating virus expressing membrane-bound immune activators like IL-2 tips the cancer-immune set point, and in combination with immune checkpoint inhibitors may offer new hope to advanced cancer patients.

## Results

### IL-2 expression from membrane-bound IL-2 constructs

**Fig. 1:**
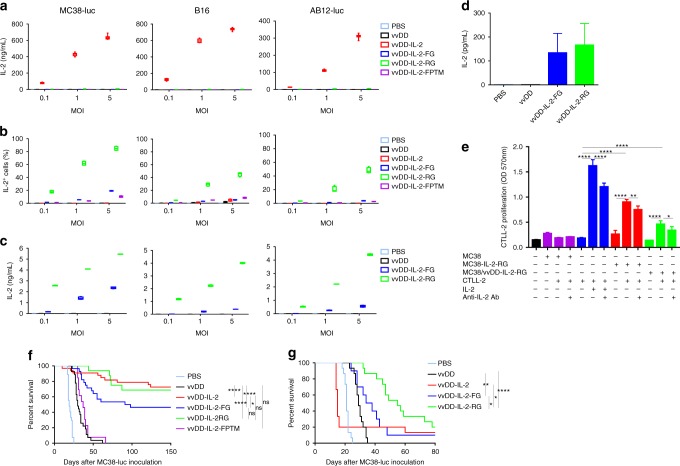
Expression of IL-2 variants and their antitumour effects. Tumour cell MC38-luc (3 × 10^5^ cells), B16 (2 × 10^5^ cells), or AB12-luc (3 × 10^5^ cells) were mock-infected or infected with vvDD, vvDD-IL-2, vvDD-IL-2-FG, vvDD-IL-2-RG, or vvDD-IL-2-FPTM at a MOIs of 0.1, 1, and 5. The culture supernatants were harvested to measure secreted IL-2 by ELISA (**a**) and the cell pellets were harvested to measure membrane-bound IL-2 either by by flow cytometry (cell surface staining) (**b**) or by ELISA after PI-PLC cleavage (**c**) 24 h post-infection. B6 mice were i.p. inoculated with 5 × 10^5^ MC38-luc cells and treated with PBS (*n* = 5), vvDD (*n* = 5), vvDD-IL-2-FG (*n* = 5), or vvDD-IL-2-RG (*n* = 6) at 2 × 10^8^ PFU per mouse 5 days post-tumour inoculation and GPI-anchored IL-2 was measured by ELISA post PI-PLC cleavage from single cells of tumour mass 5 days after treatment (2 × 10^6^ cells were incubated with 200 μL PI-PLC solution (8 unit per mL PBS) (**d**). CTLL-2 was cultured with IL-2 or MMC-treated tumour cells in the presence or absence of anti-IL-2 antibody (**e**). Data are representative of two independent experiments. B6 mice were i.p inoculated with 5 × 10^5^ MC38-luc cells and treated with PBS (*n* = 27), vvDD (*n* = 27), vvDD-IL-2 (*n* = 33), vvDD-IL-2-FG (*n* = 28), vvDD-IL-2-RG (*n* = 16), or vvDD-IL-2-FPTM (*n* = 13) at 2 × 10^8^ PFU per mouse 5 days post-tumour inoculation. Data are combined from four independent experiments (**f**). B6 mice were i.p. inoculated with 5 × 10^5^ MC38-luc cells and treated with PBS (*n* = 15), vvDD (*n* = 15), vvDD-IL-2 (*n* = 15), vvDD-IL-2-FG (*n* = 10), or vvDD-IL-2-RG (*n* = 15) at 2 × 10^8^ PFU per mouse 9 days post-tumour inoculation (**g**). Data are combined from two independent experiments. A log-rank (Mantel-Cox) test was used to compare survival rates. *P<0.05; **P<0.01; ***P<0.001; and ****P<0.0001. ns: not significant

Virus-delivered secreted IL-2 has the potential to treat established tumours in mouse models (Supplementary Fig. 1)^20^. A low level of secreted IL-2 after infection is both safe and efficacious, but the lack of control of IL-2 expression in vivo can lead to toxicity. In an attempt to reduce the severe toxic side effects caused by systemic exposure of high-dose IL-2, we used as a backbone a thymidine kinase (TK), and vaccinia growth factor (VGF) deficient oncolytic vaccinia virus (vvDD) to deliver membrane-bound IL-2 into the tumour microenvironment. This replicating virus is tumour-selective^21^ and is safe in clinical trials as an intratumoural^22^ or intravenous^23^ injection. We examined the expression of IL-2 by vvDD using the secreted form (vvDD-IL-2) and multiple techniques for membrane association, including a transmembrane domain, a glycosylphosphatidylinositol (GPI) anchor with a flexible linker, and a GPI anchor with a rigid linker (vvDD-IL-2-FPTM, vvDD-IL-2-FG, and vvDD-IL-2-RG, respectively, Supplementary Fig. 2). vvDD-IL-2-FPTM produced IL-2 fused with the murine PD-L1 transmembrane domain and a flexible linker (G_4_S)_3._ vvDD-IL-2-FG and vvDD-IL-2-RG produced IL-2 fused with the GPI anchor sequence of human CD16b, with a flexible linker (G_4_S)_3_ or a rigid linker (A(EA_3_K)_4_AAA)^24^ in between, respectively. These four viruses have similar capacity for replication and cytotoxicity in tumour cells in vitro, compared with the parental virus, vvDD (Supplementary Fig. 3). As expected, vvDD-IL-2 produced significantly more IL-2 in supernatants in vitro than the other constructs (Fig. 1a). On the contrary, the other three viruses produced only membrane-bound IL-2, though the amount of membrane-bound IL-2 was highest with the rigid linker fused to GPI (vvDD-IL-2-RG) compared to the flexible linker or the transmembrane domain (Fig. 1b and Supplementary Fig. 4). This was confirmed by the amount of IL-2 cleaved by phosphatidylinositol-specific phospholipase C (PI-PLC) from membrane associated GPI-anchored IL-2 in vitro and in vivo (Fig. 1c, d), and the amount of either secreted IL-2 or membrane-bound IL-2 was correlated to the multiplicity of infection (MOI) of viruses (Fig. 1a–c). The gene expression data showed that the viral housekeeping gene (A34R) mRNA levels were similar among the constructs, but the IL-2 mRNA levels varied significantly, suggesting that the components of chimeric proteins might impact the mRNA stability and thus also effect the amount of IL-2 displayed on the cell membrane (Supplementary Fig. 5). Whether the protein structure or mRNA stability best defines the activity of the construct is unknown. We further demonstrated that the GPI-anchored IL-2 is functional in vitro. Both the stable IL-2-RG expressing cell line (MC38-IL-2-RG) and vvDD-IL-2-RG-infected MC38 cells could stimulate the proliferation of CTLL-2, whose growth is IL-2-dependent, and this proliferation could be suppressed by IL-2 blocking antibody (Fig. 1e).

### Toxicity and antitumour effects of IL-2 constructs

**Fig. 2:**
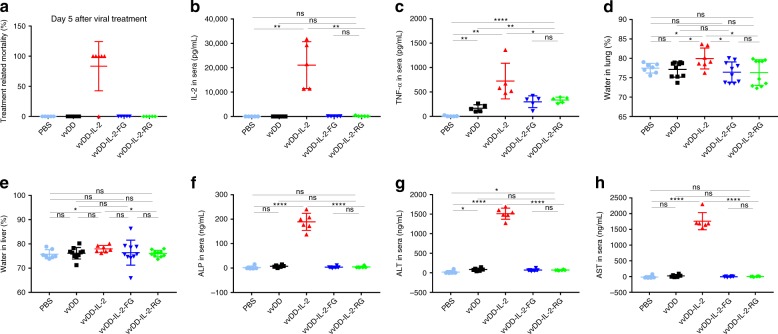
Toxicity profile of viral-delivered IL-2 variants. B6 mice were i.p. inoculated with 5 × 10^5^ MC38-luc cells and treated with PBS, vvDD, vvDD-IL-2, vvDD-IL-2-FG, or vvDD-IL-2-RG at 2 × 10^8^ PFU per mouse 9 days post-tumour inoculation. The mice that died earlier than PBS-treated mice, generally within 7 days post-viral treatment, were counted as IL-2-induced death. Five mice were used for each treatment group except vvDD-IL-2 treatment group (*n* = 6) and data are representative of two independent experiments (**a**). The treated mice were sacrificed 4 to 5 days post-treatment for blood collection to measure IL-2 (**b**) and TNF-α (**c**) in sera and to monitor pulmonary oedema (**d**) and hepatic oedema (**e**). Liver toxicity was assessed by measuring ALP, ALT, and AST in sera by ELISA (**f–h**). Five mice were used for each treatment group and data are representative of two independent experiments (**b**, **c**); data are combined from two independent experiments (**d**, **e**) and seven mice were used for PBS or vvDD-IL-2 treatment, and ten mice for other treatments, respectively. Six mice were used for each treatment group and data are representative of two independent experiments (**f–h**); *P<0.05; **P<0.01; ***P<0.001; and ****P<0.0001. ns: not significant

To evaluate the antitumour efficacy of the four viruses, we injected virus intraperitoneally at the dose of 2 × 10^8^ PFU per mouse to treat B6 mice bearing 5-day-old peritoneal murine colon cancer (MC38-luc, early-stage tumour model). The survival results demonstrated that vvDD-IL-2, vvDD-IL-2-FG, and vvDD-IL-2-RG, but not vvDD-IL-2-FPTM, elicited potent antitumour effects compared to PBS or vvDD treatment (Fig. 1f). The transmembrane domain conjugated IL-2 was ineffective, probably due to low amounts of IL-2 displayed on the cell membrane post vvDD-IL-2-FPTM infection (Fig. 1a, b and Supplementary Figs. 4 and 5). The mice treated with vvDD-IL-2 survived longer than those treated with vvDD-IL-2-FG, but not than those treated with vvDD-IL-2-RG (Fig. 1f). All the mice cured by vvDD-IL-2, vvDD-IL-2-FG, and vvDD-IL-2-RG treatment rejected a subcutaneous MC38 re-challenge (Supplementary Fig. 6a), but not an irrelevant tumour control Lewis lung cancer challenge (Supplementary Fig. 6b), which suggests that a systemic tumour-specific antitumour immune response was elicited. Occasionally, a few mice died within 1 week post vvDD-IL-2 treatment (Fig. 1f), consistent with IL-2-induced toxicity. We found that the tumour burden positively correlated with the amount of IL-2 in mouse sera after vvDD-IL-2 treatment as well as the observed level of frailty of the animals after treatment (Supplemental data Fig. 7 and observation). We, therefore, hypothesised that secreted IL-2 produced by vvDD-IL-2 treatment might be more toxic for mice with a heavy tumour burden, due to more viral replication leading to increased IL-2 expression, and a weakened clinical state. Thus, we assessed the safety and antitumour efficacy of the viruses in a 9-day-tumour-bearing mouse model (late-stage tumour model). This advanced disease model is more akin to metastatic human tumours, and is considerably more immunosuppressive with heavier tumour burden (Supplementary Fig. 8a–c) and increased immunosuppressive factor expression in the tumour microenvironment (PD-1, PD-L1, CTLA-4, TGF-β, CD105, and VEGF, Supplementary Fig. 8d). The late-stage tumour mice also have more severe oedema in the livers (Supplementary Fig. 8e). The therapeutic results in the late-stage tumour model showed that vvDD-IL-2 treatment led to a very high mortality at day 5 post-treatment (IL-2 toxicity), whereas other viruses conferred safety, and the treatments of vvDD-IL-2-FG and vvDD-IL-2-RG significantly extended the animal survival, compared to vvDD treatment. VvDD-IL-2-RG elicited a significantly improved survival compared to all other viruses (Figs. 1g and 2a). We explored the efficacy of vvDD-IL-2-RG in other tumour models, including ovarian cancer (ID8), Lewis lung cancer (LLC) and another colon cancer (CT26) with similar results (Supplemental Fig. 9a–c).

To further investigate the toxicity induced by these viruses, we measured the IL-2 in mouse sera and found that IL-2 levels were 100 times higher in sera from mice treated with vvDD-IL-2 compared to those treated with membrane-bound forms and reached an average of 21,061 pg per mL (Fig. 2b). A small amount of IL-2 was also measured in sera from mice treated with vvDD-IL-2-FG and vvDD-IL-2-RG since GPI-anchored proteins are associated with membranes more loosely than transmembrane proteins and might spontaneously release from the cell membrane due to shedding or proteolytic cleavage, notwithstanding that free GPI-anchored proteins might also transfer to other cell membranes via a process termed ‘‘cell surface painting’’^25^. Next, we examined the IL-2-induced inflammatory cytokine TNF-α in sera, a known mediator of IL-2 toxicity^26^. The result showed that vvDD-IL-2 treatment was associated with significantly elevated TNF-α (Fig. 2c). We also assessed mice for tissue oedema, a hallmark for measuring IL-2 induced vascular leak syndrome^27^. Only vvDD-IL-2 treatment induced pulmonary and hepatic oedema, as evidenced by an increase in water content in lungs and livers (Fig. 2d, e). Actually, we also found tissue oedema and increased IL-2 in sera from vvDD-IL-2-treated mice in the early-stage tumour model (Supplementary Fig. 10a–d). We further assessed the liver toxicity by measuring alkaline phosphatase (ALP), alanine transaminase (ALT), and aspartate transaminase (AST) in sera and the results showed that vvDD-IL-2 treatment led to a significantly higher ALP, ALT, and AST in sera (Fig. 2f–h). Taken together, our data demonstrates that vvDD-IL-2-FG and vvDD-IL-2-RG effectively maintains the IL-2 in the tumour microenvironment (Fig. 1d), and are, therefore, far safer than vvDD-IL-2.

### Mechanism of activity: examining the tumour microenvironment

**Fig. 3:**
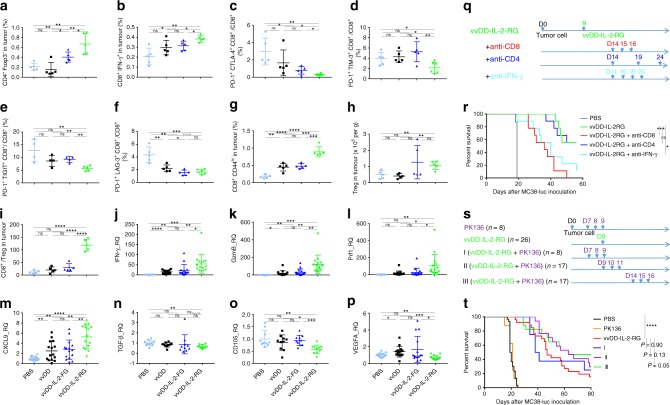
Immune status change in the tumour microenvironment post-virus treatments. B6 mice were inoculated i.p. with 5 × 10^5^ MC38-luc cells and treated with PBS, vvDD, vvDD-IL-2-FG, or vvDD-IL-2-RG at 2 × 10^8^PFU per mouse 9 days post-tumour inoculation. Tumour-bearing mice were sacrificed 5 days post-treatment and primary tumours were collected and analysed by flow cytometry to determine CD4^+^Foxp3^-^ (**a**) and CD8^+^IFN-γ^+^ T cells (**b**), exhausted CD8^+^ T cell (**c–f**), memory-phenotype T cells (CD8^+^CD44^hi^) (**g**), regulatory T cells (CD4^+^Foxp3^+^) (**h**), CD8/Treg (**i**), or by RT-qPCR to determine IFN-γ, granzyme B, perforin, CXCL9, TGF-β, CD105, and VEGF (**j–p**). Four to five mice were used for each treatment group and data are representative of two independent experiments (**a**–**i**) or combined from three independent experiments (**j–m**) or two independent experiments (**n–p**). In a separate experiment, B6 mice were i.p. inoculated with 5 × 10^5^ MC38-luc cells and treated with vvDD-IL-2-RG or PBS 9 days post-tumour inoculation. Anti-CD8 Ab (250 µg per injection), anti-CD4 Ab (150 µg per injection), anti-IFN-γ Ab (200 µg per injection) (nine mice per group) (**q**), or PK136 (300 µg per injection) (**s**), were i.p. injected into mice to deplete CD8^+^ T cells, CD4^+^ T cells or neutralise circulating IFN-γ, or NK1.1^+^ cells, and a log-rank (Mantel-Cox) test was used to compare survival rates (**r**, **t**), respectively. *P<0.05; **P<0.01; ***P<0.001; and ****P<0.0001. ns: not significant

We explored the mechanism behind the antitumour immune activity of vvDD-IL-2-RG in the profoundly immunosuppressive advanced tumour model. We investigated the immune cell profile in the tumour microenvironment and spleens using the late-stage tumour model. The percentages of activated CD4^+^Foxp3^−^ and CD8^+^IFN-γ^+^ T cells from tumours receiving vvDD-IL-2-RG treatment were increased, compared to those treated with other viruses (Fig. 3a, b). The results also showed that the percentage of total CD8^+^ cells increased and the more severely exhausted PD1^+^CTLA-4^+^CD8^+^, PD1^+^Tim-3^+^CD8^+^, PD1^+^TIGIT^+^CD8^+^, and PD1^+^LAG-3^+^CD8^+^ T cells in the tumour infiltrating CD8^+^ T-cell population decreased after vvDD-IL-2-RG treatment (Fig. 3c–f and Supplementary Fig. 11a). We examined memory CD8^+^CD44^hi^ T cells and CD3^−^NK1.1^+^ cells^28^, and the percentages of these cells from the tumour and spleen post vvDD-IL-2-RG treatment were increased compared to those treated with other viruses (Fig. 3g and Supplementary Fig. 11b–d). We also examined Tregs and found that the absolute number and percentage of CD4^+^Foxp3^+^ T cells in tumours were increased (Fig. 3h and Supplemental Fig. 11e), but the CD8^+^/Treg ratio was significantly higher after treatment with vvDD-IL-2-RG compared to other virus treatment (Fig. 3i). Next, we examined the expression of anti- and pro-tumoural immune factors in the late-stage tumour microenvironment post-virus treatment. The data showed that there were significantly more IFN-γ, Granzyme B, perforin, Th1-type chemokine CXCL9, and less TGF-β and angiogenesis markers (CD105 and VEGF) in tumours receiving vvDD-IL-2-RG treatment, compared with other virus treatments (Fig. 3j–p). We further depleted IFN-γ, CD4^+^, and CD8^+^ T cells by antibodies post vvDD-IL-2-RG treatment and the antitumour effect elicited by vvDD-IL-2-RG treatment was IFN-γ and CD8^+^ T-cell dependent, but not CD4^+^ T-cell dependent (Fig. 3q, r). To investigate the role of NK cells in vvDD-IL-2-RG treatment, we depleted NK1.1^+^ cells by antibody before, simultaneously, or after vvDD-IL-2-RG treatment. Regardless of the timing of NK cell depletion, it did not impair the therapy. On the contrary, depletion of NK1.1^+^ cells after vvDD-IL-2-RG treatment led to a slight survival benefit, compared with vvDD-IL-2-RG treatment alone, suggesting that NK cells might impede virotherapy via its natural cytotoxicity receptor NKP46^29^, which was significantly increased by vvDD-IL-2-RG treatment (Supplementary Fig. 12), and NK cell depletion might remove this impediment (Fig. 3s, t). Collectively, these data demonstrated that vvDD-IL-2-RG treatment tipped the cancer-immune set point in tumour-bearing mice from immune-suppressive to immune-favourable, which led to a better survival outcome after vvDD-IL-2-RG treatment.

### Effects of vvDD-IL-2-RG combined with checkpoint inhibition

**Fig. 4:**
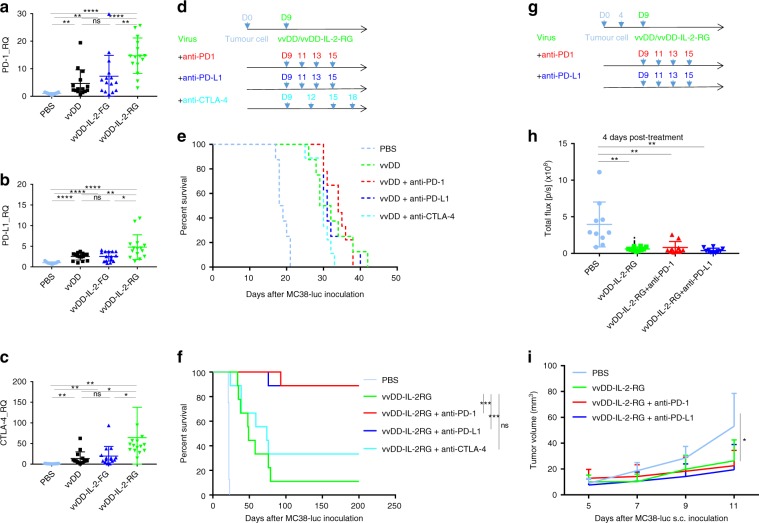
Combining vaccinia virus expressing IL-2 with immune checkpoint therapy. B6 mice were inoculated with 5 × 10^5^ MC38-luc cells and treated with PBS, vvDD, vvDD-IL-2-FG, or vvDD-IL-2-RG at 2 × 10^8^ PFU per mouse 9 days post-tumour inoculation. Tumour-bearing mice were sacrificed 5 days post-treatment and primary tumours were collected and analysed using RT-qPCR to determine the expressions of PD-1 (**a**), PD-L1 (**b**), and CTLA-4 (**c**) in the tumour microenvironment. Five mice were used for each treatment group in one experiment and data are combined from three independent experiments. In a separate experiment, B6 mice were i.p. inoculated with 5 × 10^5^ MC38-luc cells and treated with vvDD/vvDD-IL-2-RG or PBS 9 days post-tumour inoculation. Anti-PD-1 Ab (200 µg per injection), anti-PD-L1 Ab (200 µg per injection), or anti-CTLA-4 Ab (100 µg per injection) were i.p. injected into mice as scheduled (nine mice per group) (**d**), and a log-rank (Mantel-Cox) test was used to compare survival rates (**e**, **f**). In some experiments, to measure the abscopal effect, B6 mice were also s.c. inoculated with 5 × 10^5^ MC38-luc 4 days after i.p. tumour inoculation, and treated with i.p. vvDD-IL-2-RG alone or combined with α-PD-1/PD-L1 Ab as scheduled (ten mice per group) (**g**) to monitor s.c. tumour growth (**h**, **i**). *P<0.05; **P<0.01; ***P<0.001; and ****P<0.0001. ns: not significant

It is important to note that the vvDD-IL-2-RG construct induced systemic antitumour immunity, but in the case of high tumour burden this antitumour effect was unable to clear all of the disease. The tumour eventually progressed in the majority of animals, leading to their death. We hypothesise that this is due to the treatment-induced adaptive immune resistance, as evidenced by the elevated PD-1, PD-L1, and CTLA-4 expression in tumours (Fig. 4a–c)^30^. We previously reported that the rational combination of oncolytic vaccinia virus and anti-PD-L1 antibody could work synergistically to enhance therapeutic efficacy in the early-stage tumour model^31^. We, therefore, tested whether the combination of vvDD/vvDD-IL-2-RG and anti-PD-1/PD-L1 or anti-CTLA-4 antibody could improve the therapeutic effects using the late-stage tumour model. The MC38-luc-bearing mice were treated (Fig. 4d) and the survival results showed that the combination of vvDD and antibodies did not improve the survival in this late-stage model (Fig. 4e). However, the combination of vvDD-IL-2-RG and anti-PD-1/PD-L1 antibody, but not anti-CTLA-4 antibody, cured most of the advanced tumour-bearing mice (Fig. 4f). We further found that the treatment of vvDD-IL-2-RG or vvDD-IL-2-RG plus anti-PD-1/PD-L1 antibody for intraperitoneal tumour could elicit an abscopal effect on non-treated subcutaneous tumours in the flank (Fig. 4g–i and Supplementary Fig. 13a–c).

## Discussion

Cytokines, such as IL-2 have played a pivotal role in the war against multiple types of cancer, however, the clinical applications are limited due to the severe toxicities associated with high doses delivered systemically. Attempts have been made to modify IL-2 to maintain activity and minimise toxicity, with variable success^10–19^. In order to develop a safer, more effective immunotherapy using IL-2, we engineered a number of membrane-bound constructs expressed by a tumour-selective, oncolytic vaccinia virus^21^. The construct using a GPI anchor with a rigid linker to IL-2 was highly expressed on the surface of cells and functional both in vivo and in vitro. Compared to the secreted form of IL-2, the membrane-bound form maintains activity, but abrogates the associated toxicity. The combination of a replicating vaccinia virus and intratumoural expression and maintenance of IL-2 results in dramatic antitumoural activity, dependent on CD8^+^ T cells and IFN-γ. The IL-2 expression leads to a favourable immune profile in the tumour microenvironment, which correlates with the antitumour response.

Oncolytic viruses have been extensively used to deliver cytokines in order to improve the immune effects of the virus, but the systemic consequences of the released proteins has not been adequately addressed^20,32–35^. As replicating vectors become more efficient, the amount of virus infecting tumour cells increases, and circulating cytokines expressed by the virus may be toxic. This is demonstrated in our animal models, where increased tumour burden leads to increase viral replication and toxic amounts of circulating IL-2 when expressed as a secreted protein. In a vaccinia virus clinical trial, JX-594 treatment of hepatocellular cancers led to quantifiable, circulating GM-CSF levels in 69% of the patients receiving 10^9^ plaque forming units of virus^36^. These data suggest that oncolytic virus-delivered, secreted cytokines might be toxic in clinical applications. The GPI anchor and rigid linker peptide described in the current study, was successful in both maintaining IL-2 membrane association and activity, which further leads to enhanced safety and remarkable efficacy. This approach might also be effective for other cytokines or proteins.

The vvDD-IL-2-RG construct induced potent antitumour immunity, which cures the majority of mice with lower tumour burden, but not with higher tumour burden. This failure in the setting of higher tumour burden might be attributed to the treatment-induced adaptive immune resistance, as evidenced by the elevated PD-1, PD-L1, and CTLA-4 expression in tumours^30^. This resistance was relieved by the combination of vvDD-IL-2-RG and anti-PD-1/PD-L1 but not anti-CTLA-4 antibody. The lack of effect with anti-CTLA-4 antibody treatment compared to anti-PD-1/PD-L1 antibody treatment might be attributed to their different mechanisms of action. Anti-CTLA-4 antibody primarily effects CD4^+^ T cells at the priming phase of the immune response, while anti-PD-1/PD-L1 acts predominantly on exhausted T cells within the tumour, which might be essential for overcoming the more immunosuppressive microenvironment in late-stage solid tumours. It is worthwhile to point out that vvDD combined with anti-PD-L1 Ab could elicit effective antitumour activity in the early-stage tumour model^31^, but not the late-stage tumour model, which suggests that only the appropriately armed oncolytic virus could effectively modulate the more immunosuppressive tumour microenvironment, as supported by other recent studies^37,38^. Apart from combining with immune checkpoint inhibitors, the integration of other cytokines or therapeutic molecules into one vaccinia virus with our shuttle vector (pCMS1-IRES) to break adaptive immune resistance and to improve the therapeutic efficacy of vvDD-IL-2-RG in the late-stage tumour model is currently under investigation.

The complex tumour microenvironment may require an equally complex immunotherapy approach. Vaccinia virus is a multifaceted vector with a complicated immune effect. It produces a number of immunosuppressive proteins^39^, yet in humans it is rapidly cleared by the immune system. Both cellular and humoral immunity are activated and contribute to viral clearance. Even within the immunosuppressive tumour microenvironment, a replicating vaccinia virus is effectively, immunologically cleared in humans before complete clearance of the tumour^22,23^. The combination, however, of a replicating virus expressing an immune activating cytokine, with immune checkpoint blockade can affect almost all aspects of the cancer-immunity cycle^1,5,40^. This includes replicating virus-induced immunogenic cell death leading to effective antigen processing and presentation, and elicitation of danger signals to attract cytotoxic T-cells; cytokine-induced activation and expansion of those cells for immunologic bystander killing of non-infected cells; and cytokine-induced persistence of activated cells and immune checkpoint blockade to allow complete tumour eradication. In the current study, the immune microenvironment was affected in a global way, increasing the immune-stimulatory cells and proteins, and decreasing the exhausted phenotypes. This multifaceted approach may have advantages over more focused approaches for the treatment of non-immunogenic tumours.

In summary, our data demonstrate that vvDD-IL-2-RG treatment is a safe method of IL-2 delivery and treatment, creating the optimal immune microenvironment while avoiding systemic toxicity. Further, in profoundly immunosuppressive, advanced stage disease, vvDD-IL-2-RG synergises with anti-PD-1/PD-L1 antibody therapy leading to the cure of most late-stage tumours. Our data suggest that vvDD-IL-2-RG as a new form of IL-2 immunotherapy can reverse the cancer-immune set point and represent a treatment for cancers, which are to date unresponsive to immunotherapy.

## Methods

### Mice and cell lines

Female C57BL/6 (B6 in short) and BalB/c mice were purchased from The Jackson Laboratory (Bar Harbor, ME) and housed in specific pathogen-free conditions in the University of Pittsburgh Animal Facility. All animal studies were approved by the University of Pittsburgh Institutional Animal Care and Use Committee. Mouse colon cancer MC38-luc, ovarian cancer ID8-luc, and mesothelioma AB12-luc were generated by the infection of parental tumour cells with firefly luciferase-carrying lentivirus and antibiotic blasticidin selection^41^. Mouse colon cancer MC38-IL-2-RG was generated by the transfection of pLenti6-mIL-2-RG and selected with antibiotic blasticidin. Normal African green monkey kidney fibroblast CV-1, mouse lymphoblast CTLL-2, and mouse melanoma B16 and Lewis lung cancer cells were obtained from American Type Culture Collection. CTLL-2 was grown in RPMI-1640 supplemented with 10% fetal bovine serum (FBS), 1 mM sodium pyruvate, 2 mM L-glutamine, 1x penicillin/streptomycin (Invitrogen, Carlsbad, CA), and 100 unit per mL recombinant human IL-2. Other cell lines were grown in Dulbecco’s modified Eagle’s medium (DMEM) supplemented with 10% FBS, 2 mM l-glutamine, 1x penicillin/streptomycin in 37 °C, 5% CO_2_ incubator.

### Virus generation

vSC20, a *vgf* gene-deleted Western Reserve (WR) strain VV, was used as the parental virus for homologous recombination. We constructed the plasmid pCMS1-IRES carrying two multiple cloning sites separated with an IRES sequence from pLVX-IRES-ZsGreen based on a shuttle plasmid pSEM-1^42^. pCMS1-IRES was then inserted with fragments containing flexible linker or rigid linker fused with GPI anchor sequence amplified from human CD16b by PCR, resulting in plasmids pCMS1-IRES-FG or pCMS1-IRES-RG, respectively. Murine *IL-2* cDNA was inserted into pCMS1-IRES, pCMS1-IRES-FG, or pCMS1-IRES-RG to get shuttle plasmids pCMS1-IL-2, pCMS1-IL-2-FG, or pCMS1-IL-2-RG, respectively. GPI anchor sequence in pCMS1-IL-2-FG was further replaced with murine PD-L1 transmembrane domain to get shuttle plasmid pCMS1-IL-2-FPTM. The primers for plasmid cloning based on PCR are listed in Supplementary Table 1. All these shuttle vectors were used for homologous recombination of murine *IL-2* variants into the *tk* locus of vaccinia viral genome. To make the new viruses vvDD-IL-2, vvDD-IL-2-FG, vvDD-IL-2-RG, and vvDD-IL-2-FPTM, CV-1 cells were infected with vSC20 at a multiplicity of infection (MOI) of 0.1 and then transfected with the shuttle plasmids, resulting in virus mixture. Selection of the new recombinant viruses was based on expression of yellow fluorescent protein in CV-1 cells 24 h post the virus mixture infection. vvDD-YFP, or vvDD for short, a double viral gene-deleted (*tk-* and *vgf-*) VV carrying *yfp* cDNA at the *tk* locus, was the control virus for this work.

### Viral replication assays in vitro

Tumour cells were seeded at 1.0 × 10^5^ per well in six-well plates and infected with indicated viruses the next day at MOIs of 0.1, 1.0, or 10 in 1 mL medium containing 2% FBS for 2 h. Following infection, cells were added with 3 mL medium containing 10% FBS and cultured until harvest at 24, 48, and 72 h post-viral infection. The cell pellets were homogenised using a FastPrep Cell Disrupter (Model FP120; Qbiogene, Carlsbad, CA) to release virions, and the resulting cell lysates were titered on CV-1 cells to determine viral load by plaque assay.

### MTS cytotoxicity assay in vitro

Tumour cells were plated at 1.0 × 10^4^ cells per well in 96-well plates and infected with indicated viruses the next day at MOIs of 0.05, 0.1, 0.5, 1.0, and 5.0. Cell viability was determined at 48 and 72 h after infection using CellTiter 96 Aqueous Nonradioactive Cell Proliferation Assay or MTS assay (Promega, Madison, MI).

### Viral-delivered IL-2 expression in vitro

MC38-luc (3 × 10^5^), B16 (2 × 10^5^), or AB12-luc (3 × 10^5^) cells were seeded in 24-well plates overnight and infected with vvDD, vvDD-IL-2, vvDD-IL-2-FG, vvDD-IL-2-RG, or vvDD-IL-2-FPTM at MOIs of 0.1, 1, and 5 in 0.15 mL 2% FBS-containing-DMEM for 2 h. Cells were added 0.35 mL 10% FBS-containing-DMEM and cultured until harvest at 24 h post-viral infection. The culture supernatants were harvested to measure IL-2 using ELISA (BD Bioscience, San Jose, CA) and the cell pellets were applied either to measure membrane-bound IL-2 by flow cytometry, or by ELISA after cleavage of PI-PLC (Sigma, P5542; 8 unit per mL), or to extract RNA to measure IL-2 expression by quantitative reverse transcription PCR (RT-qPCR).

### CTLL-2 proliferation assay

MC38 (3 × 10^5^) cells were seeded in 24-well plates overnight and infected with vvDD-IL-2-RG at an MOI of 5 in 0.15 mL 2% FBS-containing-DMEM for 2 h. Cells were added 0.35 mL 10% FBS-containing-DMEM and cultured until harvest at 24 h post-viral infection. The virus-infected MC38, MC38, and MC38-IL-2-RG were treated with mitomycin C (MMC) (StressMarq Biosciences: SIH-246) (200 μg per mL) in 37 °C, 5% CO_2_ incubator for 2 h and washed extensively for use. CTLL-2 (7500 per well) cells were seeded in 96-well plates and cultured with either recombinant IL-2 (100 unit per mL) or MMC-treated cell prepared as mentioned (45,000 per well). For some wells, MMC-treated cells were pre-incubated with anti-mIL-2 antibody (5 μg per mL; BioLegend: #503702) for half an hour before co-culture. The proliferation of CTLL-2 was measured by MTT assay 3 days after culture.

### Rodent tumour models

B6 mice were i.p. inoculated with 5 × 10^5^ MC38-luc, 3.5 × 10^6^ ID8-luc cancer cells or BalB/c mice were i.p inoculated with 4 × 10^5^ AB12-luc, respectively, and divided into required groups at indicated day post-tumour cell inoculation according to tumour size based on live animal IVIS imaging, performed using a Xenogen IVIS 200 Optical In Vivo Imaging System (Caliper Life Sciences, Hopkinton, MA). Grouped mice were i.p. injected with indicated viruses, antibodies, the combinations, or PBS. In some experiments, anti-CD8 Ab (clone 53-6.7; Bio X Cell; 250 µg per injection), anti-CD4 Ab (clone GK1.5, Bio X Cell; 150 µg per injection), anti-NK1.1 Ab (clone PK136, Bio X Cell; 300 µg per injection), or anti-IFN-γ Ab (clone XMG1.2, Bio X Cell; 200 µg per injection) were i.p. injected into mice to deplete CD8^+^ T cells, CD4^+^ T cells, or NK1.1^+^ cells or neutralise circulating IFN-γ, respectively. Anti-PD-1 Ab (clone J43; Bio X Cell; 200 µg per injection), anti-PD-L1 Ab (clone 10 F.9G2; Bio X Cell; 200 µg per injection), anti-CTLA-4 Ab (clone 9D9; Bio X Cell; 100 µg per injection) were i.p. injected into mice for combination therapy. In some experiments, B6 mice were i.p. inoculated with 5 × 10^5^ MC38-luc at day 0, and s.c. injected with 5 × 10^5^ MC38-luc at day 4 on right flanks. In some experiments, mice were sacrificed to harvest all the individual peritoneal tumour nodules for weight and photographs, and to harvest spleens at indicated time points.

For subcutaneous tumour models, B6 or BalB/c mice were s.c. injected with 1 × 10^6^ Lewis lung cancer (LLC) or 1 × 10^6^ CT26 cells at right flanks, respectively, and intratumourally treated with vvDD, vvDD-IL-2-RG, and PBS.

MC38-luc-tumour-bearing B6 mice treated with indicated vaccinia viruses, which survived longer than 150 days, were subcutaneously challenged with 5 × 10^5^ MC38 or 1 × 10^6^ Lewis lung cancer cells per mouse. Naive B6 mice also received the same dose tumour challenge as a control. The subcutaneous tumour size was measured using an electric calliper in two perpendicular diameters.

### Assessment of treatment-related toxicity

Virus-treated mice were sacrificed 4 to 5 days post-treatments for collection of blood, lungs, kidneys, and spleens. Blood samples were kept for 2 h at room temperature and sera were separated by centrifugation to measure IL-2 and TNF-α, Alkaline phosphatase (ALP), alanine transaminase (ALT), and aspartate transaminase (AST) using commercialised kits (BD Biosciences, BioLegend, and G-BIOSCIENCES, respectively), according to the vendors’ instructions. Water content was used to monitor tissue oedema. Briefly, wet tissue was weighed and dehydrated overnight over 100 °C in a chemical hood. The weight difference between wet tissues and dry tissues was calculated.

### Flow cytometry

Collected tumour tissues were weighed and incubated in RPMI-1640 medium containing 2% FBS, 1 mg per mL collagenase IV (Sigma: #C5138), 0.1 mg hyaluronidase (Sigma: #H6254), and 200U DNase I (Sigma: #D5025) at 37 °C for 1–2 h to make single cells. In vitro virus-infected cells or single cells from tumour tissues or spleens were blocked with α-CD16/32 Ab (clone 93, eBioscience: #14-0161-85; 1:1000) and then stained with antibodies against mouse CD45 (PerCP-Cy5.5 or FITC, clone: 30-F11, BioLegend: #103132 or 103108; 1:300), CD4 (APC, clone: RM4-5, eBioscience: #17-0042-81; 1:300), Foxp3 (PE, clone: FJK-16s, eBioscience: #12-5773-82; 1:100), CD8 (PE or APC, clone: 53–6.7, eBioscience: #12-0081-85 or 17-0081-83; 1:300), PD-1 (PerCP-Cy5.5, clone: 29F.1A12, BioLegend: 135208; 1:300), CTLA-4 (APC, clone: UC10-4B9, eBioscience: #17-1522-82; 1:300), LAG-3 (PE, clone: C9B7W, BioLegend: #125208; 1:300), TIM-3 (Biotin-TIM-3, clone RMT3-23, BioLegend: #119720; 1:300 + PE-SA, eBioscience: #12-4317-87; 1:1000), TIGIT (Biotin-TIGIT, clone 1G9, BioLegend: #142113; 1:300 + PE-SA, eBioscience: #12-4317-87; 1:1000), CD44 (FITC, clone: IM7, eBioscience: #11-0441-82; 1:300), IFN-γ (APC, clone: XMG1.2, eBioscience: #17-7311-82; 1:100), CD3 (FITC, clone: 17A2, eBioscience: #11-0032-82; 1:300), NK1.1 (PE, clone: PK136, BD Biosciences: #553165; 1:300), and IL-2 (Biotin-IL-2, clone: JES6-5H4, BioLegend: #503804; 1:300; + APC-SA, eBioscience: #17-4317-82, 1:1000). The intracellular staining kit for Foxp3 and IFN-γ staining was purchased from BioLegend. Samples were collected on BD Accuri C6 cytometer, and data were analysed using BD Accuri C6 cytometer software.

### Quantitative reverse transcription PCR

Total RNA was extracted from viral-infected cells or tumour tissues using the RNeasy Kit (Qiagen, Valencia, CA). One microgram of RNA was used for cDNA synthesis, and 25 to 50 ng of subsequent cDNA was used to conduct mRNA expression analysis by TaqMan analysis on the StepOnePlus system (Life Technologies, Grand Island, NY). All the primers for the analysis were purchased from Thermo Fisher Scientific (Waltham, MA). The gene expression was normalised to the housekeeping gene HPRT1 and expressed as fold increase (2^−ΔCT^), where ΔCT = CT _(Target gene)_ – CT _(HPRT1)_.

### Statistics

Statistical analyses were performed using unpaired Student’s *t*-test (GraphPad Prism version 7). Data are means ± SD. Animal survival is presented using Kaplan–Meier survival curves and was statistically analysed using a log-rank test (GraphPad Prism version 7). Values of *P* < 0.05 were considered statistically significant, and all *P*-values were two sided. In the figures, the standard symbols were used: *P<0.05; **P<0.01; ***P<0.001; and ****P <0.0001.

## Electronic supplementary material

Supplementary Information

Peer Review File

## Data Availability

All data are available from the corresponding author upon reasonable request.
