# Supplementary material for: Modifying the cancer-immune set point using vaccinia virus expressing re-designed interleukin-2
Source: Nat Commun. 2018 Nov 8;9:4682. doi: 10.1038/s41467-018-06954-z (PMC6224581; doi:10.1038/s41467-018-06954-z)
Supplement: Supplementary file 1 — Supplementary Information [file 41467_2018_6954_MOESM1_ESM.pdf]

Modifying the cancer-immune set point using vaccinia virus expressing re-designed interleukin-2

Liu et al.

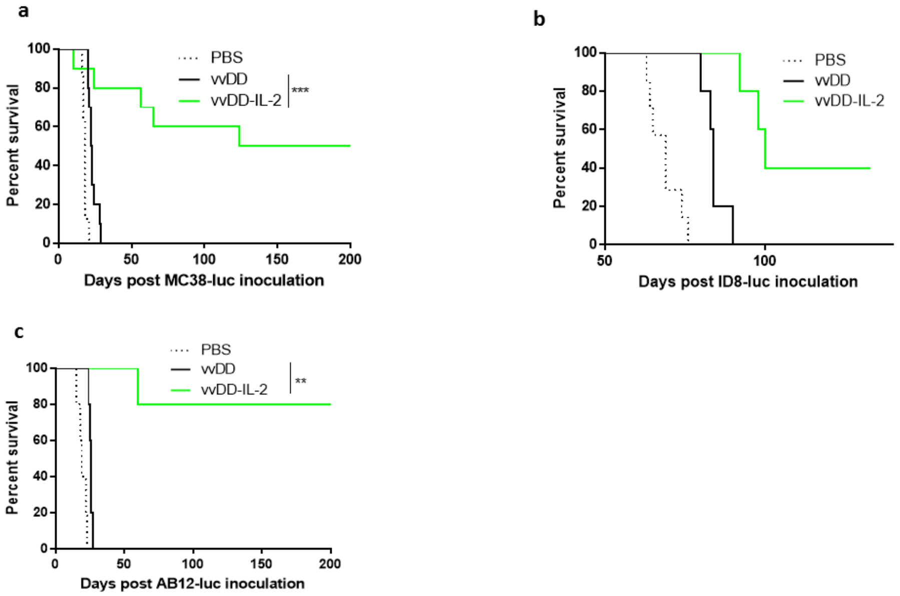

Supplementary Figure 1 | Antitumour effect of vaccinia-delivered secreted IL-2. B6 mice were inoculated i.p. with 5×10^5 MC38-luc cells (ten mice per group) (**a**) or 3.5×10^6 ID8-luc (five mice per group) (**b**) or BalB/c mice were i.p. inoculated with 4×10^5 AB12-luc (five mice per group) (**c**) and treated with PBS, vvDD, and vvDD-IL-2 at 2×10^8 PFU per mouse for MC38-luc model and 1×10^8 PFU per mouse for AB12-luc model five days post-tumour inoculation or at 2×10^8 PFU per mouse for ID8-luc model six days post-tumour inoculation. A log-rank (Mantel-Cox) test was used to compare survival rates. * $P<0.05$; ** $P<0.01$; *** $P<0.001$; and **** $P<0.0001$.

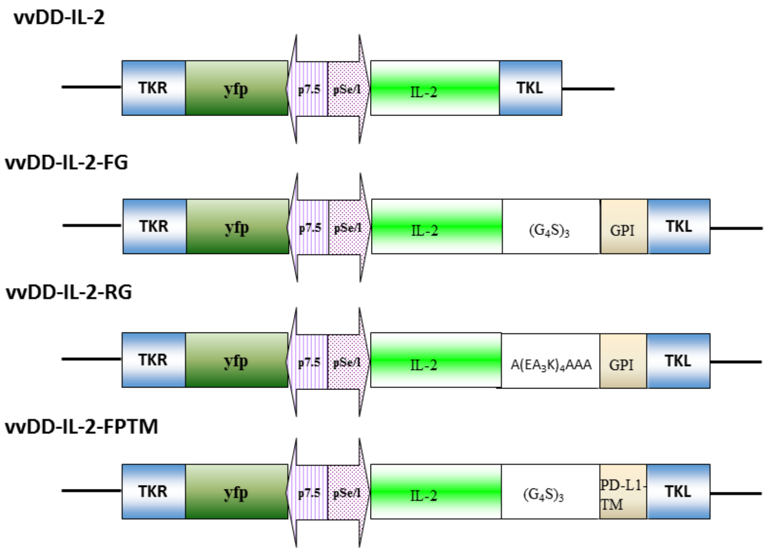

Supplementary Figure 2 | Schematic diagram of viral IL-2 variants. vvDD-IL-2, vvDD-IL-2-FG, vvDD-IL-2-RG, and vvDD-IL-2-FPTM were generated by homologous recombination of murine *IL-2* variants into the *tk* locus of vaccinia viral genome, carrying secreted IL-2, IL-2-flexible linker $(G_4S)_3$-GPI anchor sequence amplified from human CD16b, IL-2-rigid linker $A(EA_3K)_4AAA$-GPI anchor sequence amplified from human CD16b, and IL-2-flexible linker $(G_4S)_3$-murine PD-L1 transmembrane domain, respectively.

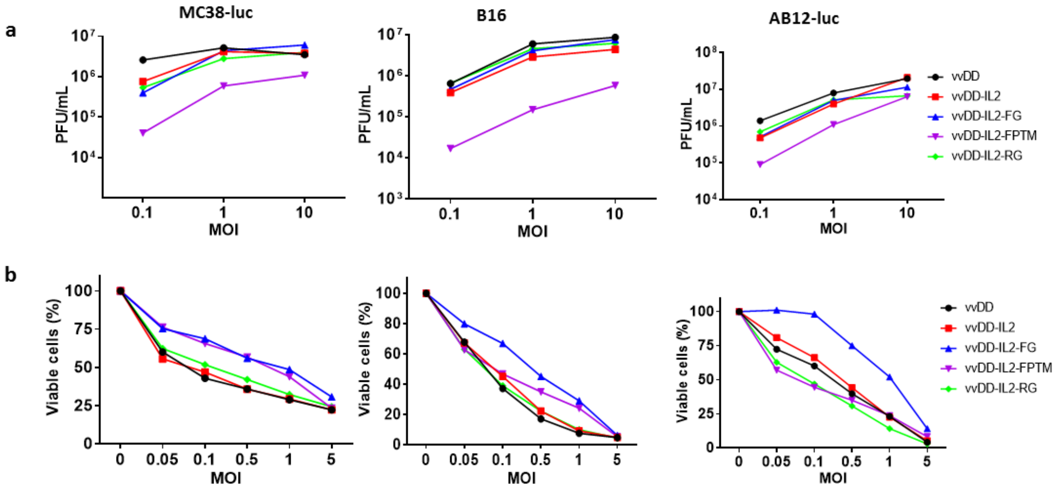

Supplementary Figure 3 | Viral replication and cytotoxicity *in vitro*. MC38-luc, B16, and AB12-luc tumour cells were mock-infected or infected with the indicated viruses. The production of viral progeny from infected cancer cells at 48 h post-infection was determined by plaque assay (**a**) or the viability of viral infected cells was determined by MTS assay (**b**). Data are representative of two independent experiments.

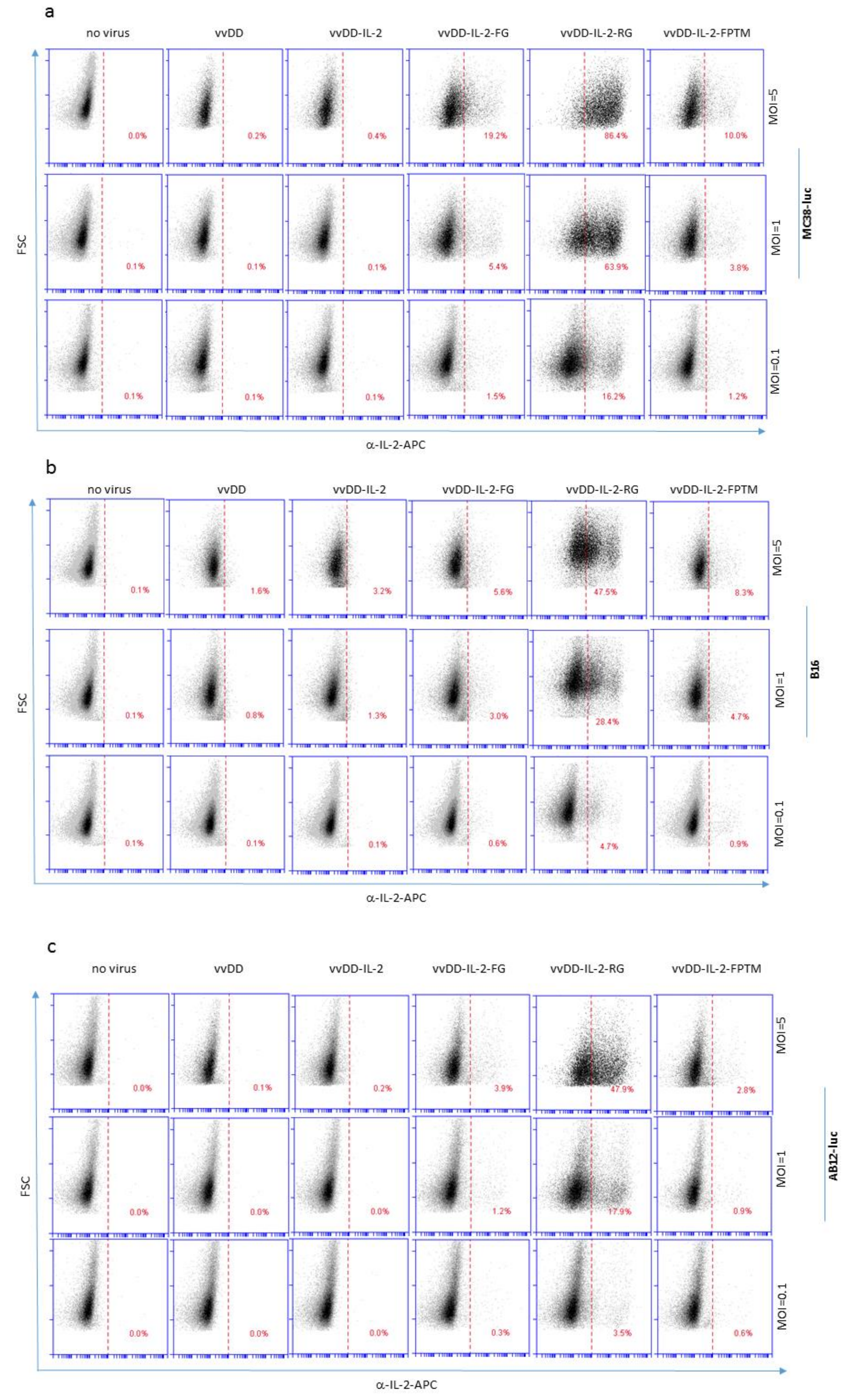

Supplementary Figure 4 | Viral delivered IL-2 expression in tumour cells. Tumour cell

MC38-luc (3×10^5 cells) (**a**), B16 (2×10^5 cells) (**b**), or AB12-luc (3×10^5 cells) (**c**) were mock-infected or infected with vvDD, vvDD-IL-2, vvDD-IL-2-FG, vvDD-IL-2-RG, or vvDD-IL-2-FPTM at a MOIs of 0.1, 1 and 5. The cell pellets were harvested and to measure membrane-bound IL-2 by by flow cytometry (cell surface staining).

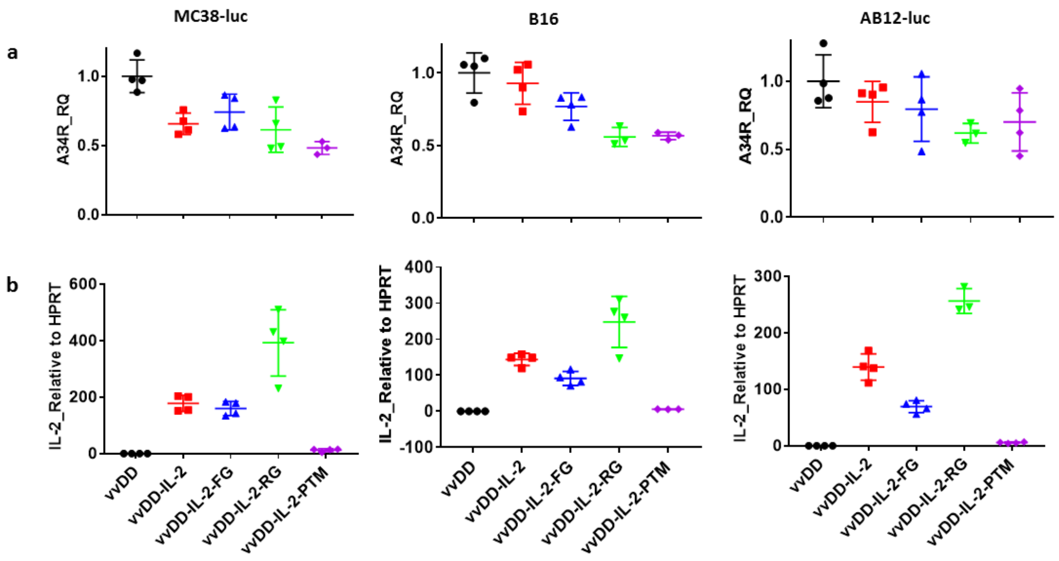

Supplementary Figure 5 | Viral delivered IL-2 expression in tumour cells. Tumour cell MC38-luc (3×10^5 cells), B16 (2×10^5 cells), or AB12-luc (3×10^5 cells) were mock-infected or infected with vvDD, vvDD-IL-2, vvDD-IL-2-FG, vvDD-IL-2-RG, or vvDD-IL-2-FPTM at a MOI of 1. The infected cells were harvested 24 h post-infection for RNA purification. Purified total RNAs were subject to RT-qPCR for quantitative detection of A34R mRNA (viral housekeeping gene) (**a**) or IL-2 (**b**). Data are representative of two independent experiments.

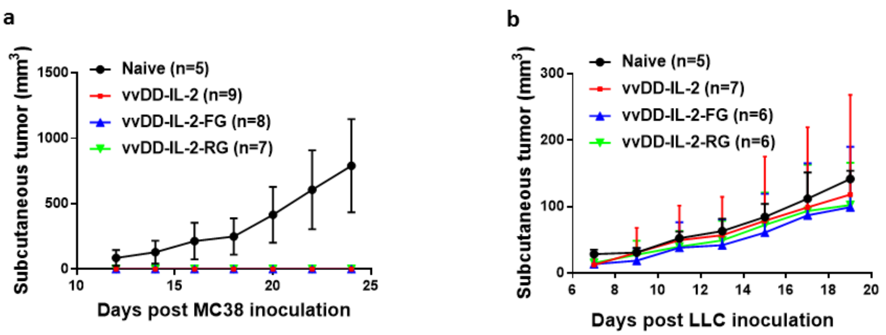

Supplementary Figure 6 | Systemic antitumour immunity elicited by viral IL-2 variants. Five-day old MC38-luc-bearing B6 mice treated with the indicated vaccinia viruses. Mice which survived more than 150 days, were subcutaneously challenged with 5×10^5 MC38 or 1×10^6 Lewis lung cancer cells per mouse. Naïve B6 mice received the same dose of tumour cells as a control. The growth curves of primary tumours are shown.

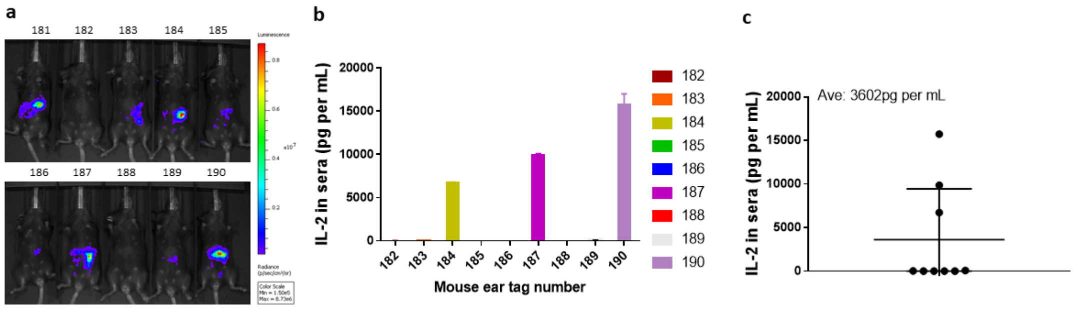

Supplementary Figure 7 | Correlation of vaccinia virus-delivered IL-2 and tumour burden in the early-stage tumour model. B6 mice were i.p. inoculated with 5×10^5 MC38-luc cells. Five days later, these mice were imaged (**a**) and i.p. treated with vvDD-IL-2 (2e8 PFU per m). The sera were collected from treated mice five days post-treatment to measure IL-2 in sera by ELISA and the IL-2 in sera was shown by individual (**b**) or pooled (**c**). Mouse ET181 was dead at day 5 post-treatment.

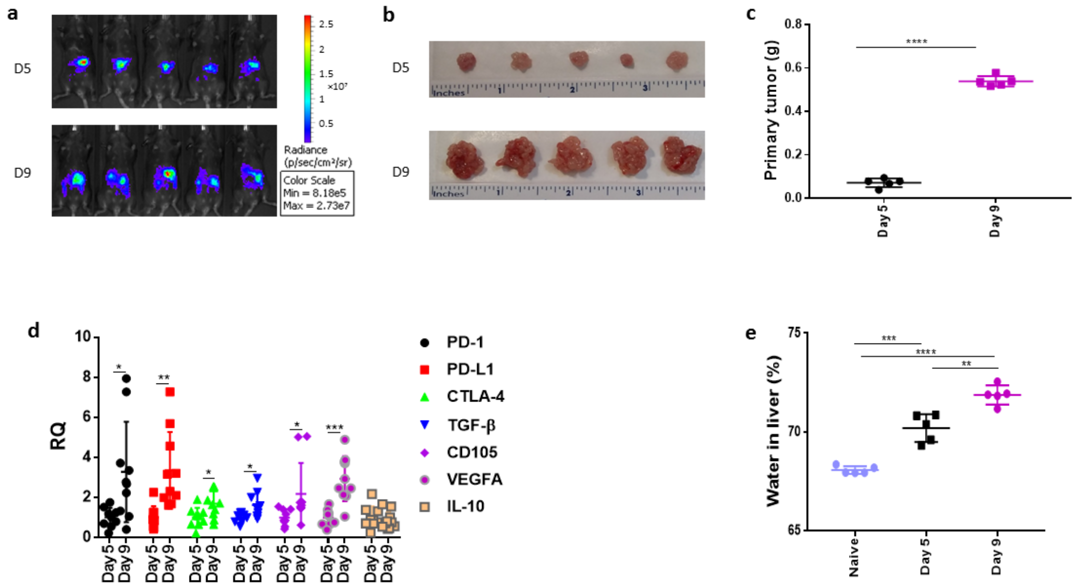

Supplementary Figure 8 | Comparison of the immunosuppressive environments of early and late-stage tumour-bearing mice. B6 mice were i.p. inoculated with 5×10^5 MC38-luc cells and imaged with a Xenogen IVIS 200 Optical In Vivo Imaging System at day 5 and day 9 post-tumour inoculation (**a**). Tumour tissues were harvested five days or nine days post-tumour inoculation. Primary tumours were photographed (**b**), weighed (**c**), and applied to extract RNA for RT-qPCR to determine the expression of immunosuppressive factors (**d**). Livers were harvested, weighed, and dried to measure water content to determine hepatic oedema (**e**) (n=5). Five mice were used for each group and data are representative of two independent experiments (**a**-**c**, **e**); nine mice were analysed for the day 5 tumour model and ten mice for the day 9 tumour model in RT-qPCR assay and data are combined from two independent experiments (**d**). * P<0.05; ** P<0.01; *** P<0.001; and **** P<0.0001.

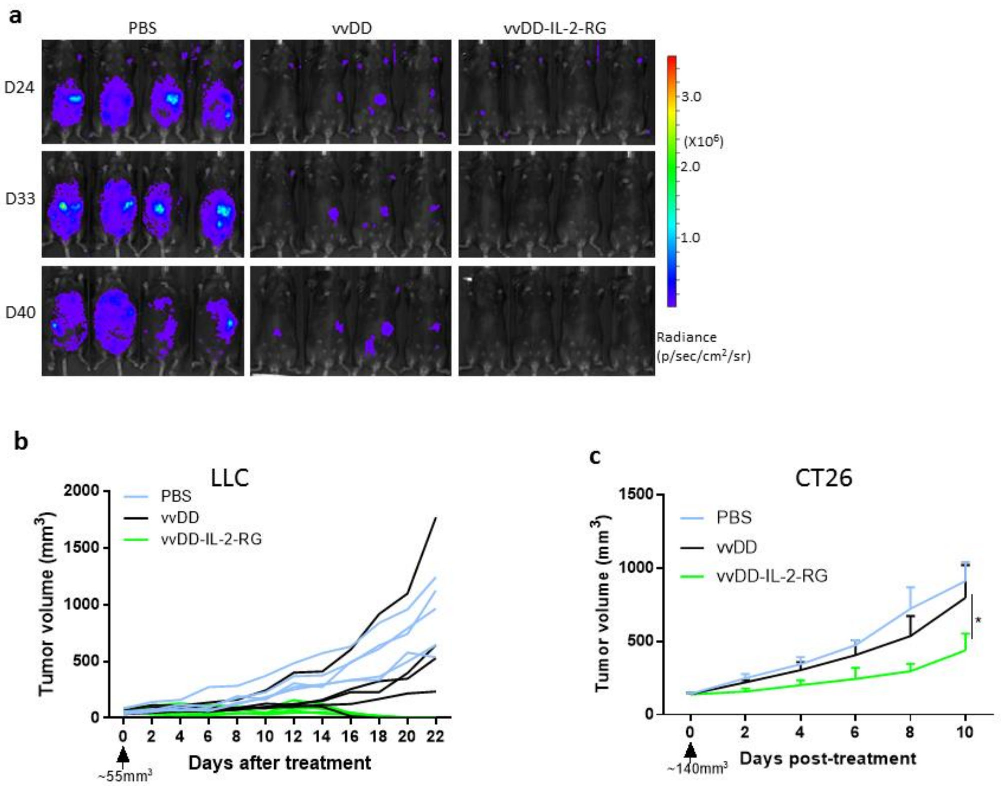

Supplementary Figure 9 | Therapeutic effect of vvDD-IL-2-RG in multiple tumour model. B6 mice were i.p. inoculated with 3.5×10^6 ID8-luc cells and treated with PBS, vvDD, or vvDD-IL-2-RG at 2×10^8 PFU per mouse six days post-tumour inoculation and tumour growth was shown in (**a**). B6 or BalB/c mice were s.c. inoculated with 1×10^6 LLC or CT26 cells and intra-tumoural treated with PBS, vvDD, or vvDD-IL-2-RG at 1×10^8 PFU per mouse at the indicated tumour volume and primary tumour growth was shown in (**b, c**), respectively.

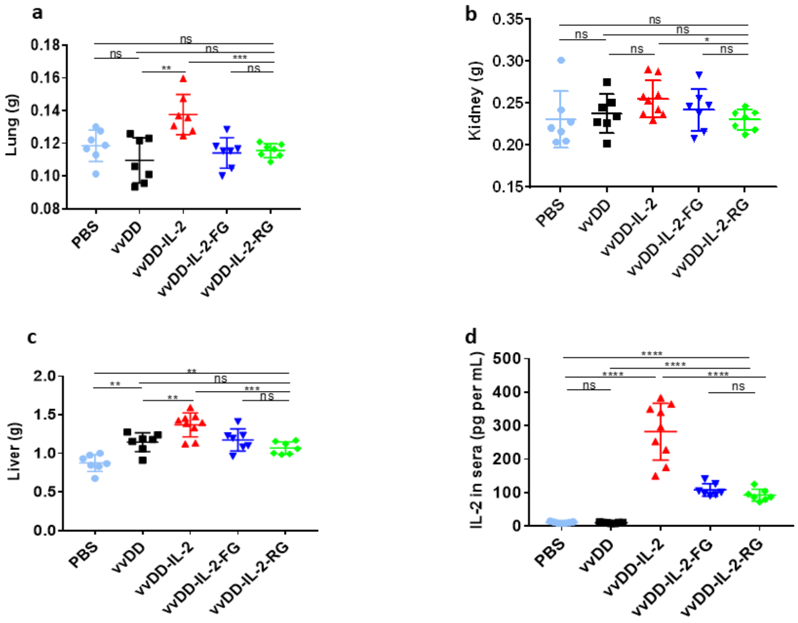

Supplementary Figure 10 | Toxicity profile of vaccinia virus-delivered IL-2 in the early-stage tumour model. B6 mice were i.p. inoculated with $5{\times}10^5$ MC38-luc cells and treated with vvDD, vvDD-IL-2, vvDD-IL-2-FG, vvDD-IL-2-RG at $2{\times}10^8$ PFU per mouse, or PBS five days post-tumour inoculation. The treated mice were sacrificed eight days post-treatment to measure water content (oedema) in lung (**a**), kidney (**b**), and liver (**c**), or to harvest blood to measure IL-2 (**d**) in sera. Data are combined from two independent experiments and seven mice were used for each group except nine mice used for vvDD-IL-2 treatment group in (**b-d**). * P<0.05; ** P<0.01; *** P<0.001; and **** P<0.0001. ns: not significant.

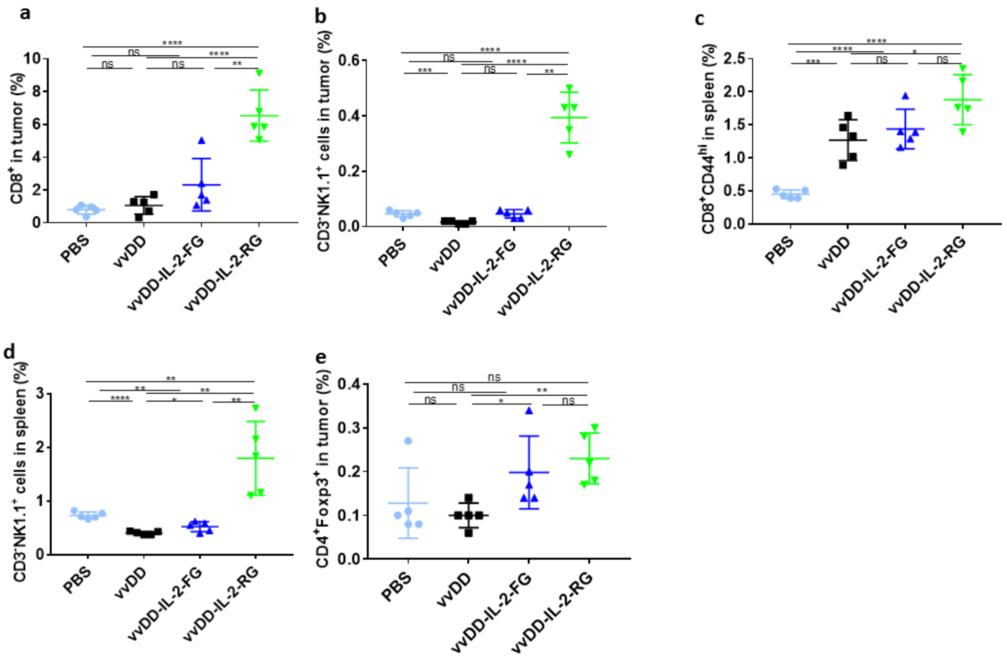

Supplementary Figure 11 | Memory-phenotype CD8^{+} T cells,NK cells and Treg cells in nine-day-tumour-bearing mice. B6 mice were i.p. inoculated with 5×10^{5} MC38-luc cells and treated treated with PBS, vvDD, vvDD-IL-2-FG, or vvDD-IL-2-RG at 2×10^{8} PFU per mouse nine days post-tumour inoculation (n=5). Primary tumours and spleens were harvested five days post treatment and applied to make single cells and stained to determine CD8^{+} in tumour (**a**), CD8^{+}CD44^{hi} in spleen (**b**), CD3^{-}NK1.1^{+} in tumour (**c**) and spleen (**d**), and Treg cells in tumour (**e**). Data are representative of two independent experiments. * *P*<0.05; ** *P*<0.01; *** *P*<0.001; and **** *P*<0.0001. ns: not significant.

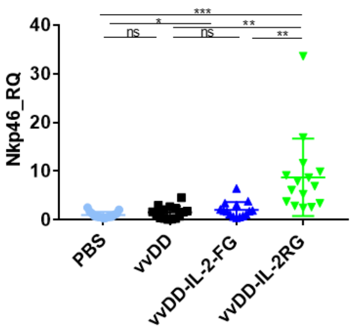

Supplementary Figure 12 | Elevated NKP46 expression in nine-day-tumour-bearing mice post-viral treatment. B6 mice were i.p. inoculated with $5{\times}10^5$ MC38-luc cells and treated with PBS, vvDD, vvDD-IL-2-FG, or vvDD-IL-2-RG at $2{\times}10^8$ PFU per mouse nine days post-tumour inoculation. Primary tumours were harvested five days post treatment and applied to extract RNA for RT-qPCR to determine the expression of NKP46. Data are combined from three independent experiments (n=15). * *P*<0.05; ** *P*<0.01; *** *P*<0.001; and **** *P*<0.0001. ns: not significant.

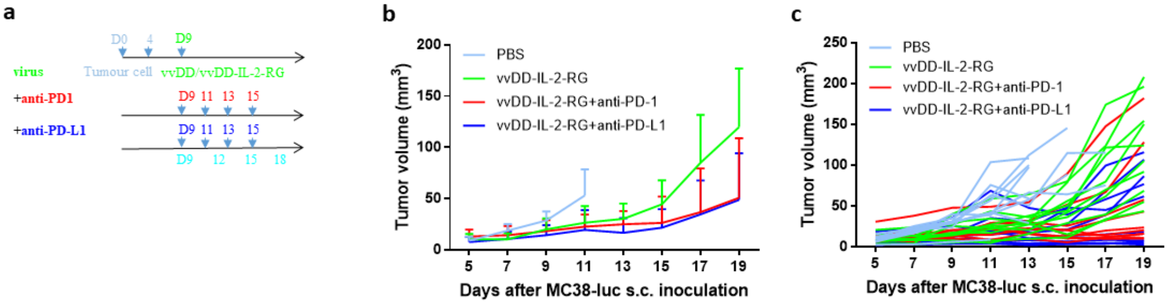

Supplementary Figure 13 | Abscopal effect of vvDD-IL-2-RG alone or combined with PD-1/PD-L1 blockade. B6 mice were i.p. inoculated with 5×10^5 MC38-luc cells at day 0 and s.c. inoculated with 5×10^5 MC38 at day 4. These tumour-bearing mice were treated with vvDD/PBS or vvDD-IL-2-RG alone or combined with anti-PD-1 Ab (200 μg per injection), anti-PD-L1 Ab (200 μg per injection) as scheduled (**a**), and the subcutaneous tumour growth was shown in pooled (**b**) or individual (**c**).

Supplementary Table 1

**Primer**	**PCR Template**	**Resulting Plasmid**
p1:5’gcgggtcgacatatggcgccatgcattataaggcgcgcccgcccctctccctccc3’<br><br>p2:5’ggccgttaacttaagagctcatttaaatcctgcagggccggccattatcatcgtg3’	pLVX-IRES-ZsGreen	pCMS1-IRES
p3:5’ggccggccggccggtggcggtgggagcggtggtgggggttccggaggcggaggg3’<br><br>p4:5’ggttccggaggcggagggtcggtgtcaaccatctcatcattctc3’<br><br>p5:5’gcgcgttaactcaaatgtttgtcttcacagag3’	Human CD16b cDNA	pCMS1-IRES-FGPI
p6:5’ggccggccggccgctgaagctgccgcaaaagaggccgctgcgaaggaggccgcggctaag3’<br><br>p7:5’gaggccgcggctaaggaggcggcagctaaagctgcagccgtgtcaaccatctcatcattc3’<br><br>p5:5’gcgcgttaactcaaatgtttgtcttcacagag3’	Human CD16b cDNA	pCMS1-IRES-RGPI
p8:5’ggcggtcgacatgtacagcatgcagctcg3’<br>p9:5’ccgcggcgcgccttattgagggcttgttgag3’	Murine IL-2 cDNA	pCMS1-IL-2-IRES
p8:5’ggcggtcgacatgtacagcatgcagctcg3’<br><br>p10:5’ccgcggccggcccttgagggcttgttgag3’	Murine IL-2 cDNA	pCMS1-IL-2-FG
p8:5’ggcggtcgacatgtacagcatgcagctcg3’<br><br>p10:5’ccgcggccggcccttgagggcttgttgag3’	Murine IL-2 cDNA	pCMS1-IL-2-RG
p3:5’ggccggccggccggtggcggtgggagcggtggtgggggttccggaggcggaggg3’<br><br>p11:5’ccggaggcggtgggtcgcactgggtgcttctgggatc3’<br><br>p12:5’cgcgttaactaccccaagaagaggaggaccgtggacactacaatgaggaacaacaggatg3’	Murine PD-L1 cDNA	pCMS1-IL-2-FPTM
p13:5’ ggcgggatccatgtacagcatgcagctcg3’3’<br><br>p14: 5’gcgcctcgagtcaaatgtttgtcttcacagag3’	pCMS1-IL-2-RG	plenti6-IL-2-RG
