# Supplementary material for: Modifying the cancer-immune set point using vaccinia virus expressing re-designed interleukin-2
Source: Nat Commun. 2018 Nov 8;9:4682. doi: 10.1038/s41467-018-06954-z (PMC6224581; doi:10.1038/s41467-018-06954-z)
Supplement: Supplementary file 2 — Peer Review File [file 41467_2018_6954_MOESM2_ESM.pdf]

Editorial Note: This manuscript has been previously reviewed at another journal that is not operating a transparent peer review scheme. This document only contains reviewer comments and rebuttal letters for versions considered at Nature Communications. Mentions of prior referee reports have been redacted.

REVIEWERS' COMMENTS:

Reviewer #1 (Remarks to the Author):

I have reviewed the revisions made by the authors and in my opinion the additional data and text that has been added addresses all of the concerns about the manuscript that were originally made. I think this is an important paper that will impact the field of therapeutic viruses.

Reviewer #2 (Remarks to the Author):

This is a review of the revised manuscript initially submitted to [Redacted]. I found that the authors have adequately addressed reviewers' comments and the manuscript is suitable for a publication in Nature Communications.
